# ConvCGP: A convolutional neural network to predict genetic values of agronomic traits from compressed genome‐wide polymorphisms

**DOI:** 10.1002/tpg2.70223

**Published:** 2026-04-19

**Authors:** Tanzila Raihan, Chyon Hae Kim, Hiroyuki Shimono, Akio Kimura, Hiroyoshi Iwata

**Affiliations:** ^1^ Graduate School of Agricultural and Life Sciences The University of Tokyo Tokyo Japan; ^2^ Department of Systems Innovation Engineering, Graduate School of Science and Engineering Iwate University Morioka Japan; ^3^ Crop Science Laboratory, Faculty of Agriculture Iwate University Morioka Japan; ^4^ Agri‐Innovation Center Iwate University Morioka Japan

## Abstract

The growing size of genome‐wide polymorphism data in animal and plant breeding has raised concerns regarding computational load and time, particularly when predicting genetic values for target traits using genomic prediction. Several deep learning and conventional methods, including dimensionality reduction techniques such as principal component analysis (PCA) and autoencoders, have been proposed to address these challenges by selecting subsets of polymorphisms or compressing high‐dimensional data for predictive analysis. However, these methods are often computationally intensive and time‐consuming. A major challenge in applying deep‐learning models directly to high‐dimensional genomic data is the substantial computational cost and time required for hyperparameter tuning and model training. To address these limitations, we propose a novel deep learning framework, *C*ompression‐based *G*enomic *P*rediction using *Conv*olutional Neural Networks (ConvCGP), that integrates autoencoder‐based nonlinear compression with convolutional neural network–based prediction in an end‐to‐end trainable pipeline. This method reduces data to a compact latent representation that retains meaningful information for prediction, thereby significantly reducing storage needs and computational load. We applied ConvCGP to high‐dimensional rice datasets for agronomic trait prediction and further tested it on maize, which is large in scale. The results show that ConvCGP maintained prediction accuracy comparable to models trained on uncompressed data, even under extreme compression where only 2% of the original features were retained. This demonstrates that ConvCGP not only scales effectively to massive datasets but also preserves predictive information under drastic dimensionality reduction. Moreover, ConvCGP consistently outperformed PCA‐based models, genomic best linear unbiased prediction, LASSO (least absolute shrinkage and selection operator), support vector machine, and other methods, establishing it as a powerful, efficient, and scalable solution for modern genomic prediction.

## Core Ideas

- The rapid growth of genome‐wide data increases computation time for trait prediction in breeding programs.
- Autoencoders compress high‐dimensional data to reduce computational load.
- Convolutional neural networks (CNNs) improve prediction accuracy when trained on compressed genotype data.
- The combined model outperforms standard machine learning and principal component analysis (PCA)‐based methods.
- This method improves efficiency in large‐scale crop genomic prediction tasks.

## Plain Language Summary

Predicting plant traits using genomic data is increasingly important in agriculture. However, the size of genetic datasets has grown rapidly, making it hard to process the data efficiently. To tackle this, we developed a new deep learning method that combines convolutional neural networks (CNNs) with autoencoders. Autoencoders reduce the size of the genetic data, making it easier to process, while CNNs help in predicting plant traits. We tested this approach on large‐scale rice and maize genetic data to predict important agronomic traits. Compared to other machine learning and data compression methods, our model made more accurate predictions while also reducing the time and computing power needed. This research shows how combining deep learning tools can help make better use of large genomic datasets in crop breeding.

**Abbreviations**
Adam: adaptive moment estimation
BayesB: Bayesian variable selection
C7AIR: Cornell‐IR LD Rice Array
CNN: convolutional neural network
DeepCGP: deep learning compression‐based genomic prediction
DL: deep learning
GBLUP: genomic best linear unbiased prediction
GS: genomic selection
GWAS: genome‐wide association studies
HDRA: high‐density rice array
Lasso: least absolute shrinkage and selection operator
MLP: multilayer perceptron
PCA: principal component analysis
ReLU: rectified linear units
RF: random forest
SNP: single nucleotide polymorphisms

## 1 INTRODUCTION

Accurately estimating and predicting the genetic values underlying target traits is a central and ongoing challenge in plant and animal breeding (Lourenço et al., 2024). Genomic selection (GS) (Meuwissen et al., 2001), which uses genome‐wide marker information to predict genetic values and to select superior genotypes (individuals or lines), greatly enhances the speed and efficiency of breeding (Hickey et al., 2017). Initially, GS relied on genotyping arrays, but with the advent of next‐generation sequencing (NGS) (Marudamuthu et al., 2023), the resulting data have become increasingly high dimensional. Managing and leveraging these high‐dimensional data for tasks such as building prediction models remain significant challenges. As the number of samples (plants and animals) used in GS continues to grow, addressing these challenges will become further critical.

Various strategies for genomic prediction have been proposed, regardless of the type of genomic data. Among them, deep learning (DL) methods have attracted significant attention in computational biology (Angermueller et al., 2016; O. Montesinos‐López et al., 2021). DL has been applied in genome‐wide association studies (GWASs)to identify single nucleotide polymorphism (SNP) interactions (Uppu et al., 2016; Wang & Wu, 2023) and to classify genomic variants (Liang et al., 2016). For example, DeepGS, an ensemble of convolutional neural network (CNN) (Krizhevsky et al., 2012) and ridge regression best linear unbiased prediction, has been used to predict genetic values from imputed SNPs (Ma et al., 2018), whereas a simple dense neural network has been utilized for genotype‐by‐sequencing data (A. Montesinos‐López et al., 2018). Abdollahi‐Arpanahi et al. (2020) compared the predictive performance of two DL methods (multilayer perceptron [MLP] and CNN), two ensemble methods (random forest [RF] and gradient boosting), and two parametric methods (genomic best linear unbiased prediction [GBLUP] and Bayesian variable selection) using real and simulated datasets. They found that DL methods only slightly outperformed parametric methods in large datasets. In these genotypic value prediction tasks, CNNs can capture spatial information from raw sequencing reads or genomic variants without the need for feature engineering. Recent studies have also explored CNN‐based frameworks for extracting informative patterns from high‐dimensional biological signals, further validating the generalizability of CNN in omics applications (Yuan et al., 2025). For instance, transfer learning‐assisted CNN models have shown strong performance in biological structure prediction using 3D genomic or phenotypic data (Yeoh et al., 2023). However, these DL methods still face challenges in fully addressing the complexities of high‐dimensional data analysis.

Recently, Nazzicari and Biscarini proposed a dimensionality reduction method using kinship matrices to compactly represent SNP genotype data. Multiple kinship matrices were stacked and processed using a two‐dimensional (2D)‐CNN for genetic value prediction (Nazzicari & Biscarini, 2022). In a similar approach, Kick et al. applied principal component analysis (PCA) for dimensionality reduction, feeding the data into a multimodal DL model. They found that the performance of multimodal DL was comparable to that of GBLUP (Kick et al., 2023). Although stacking kinship matrices and PCs helps reduce the dimensionality of dataset, these methods may lose some fine‐grained genetic information during compression, leading to inconsistent prediction performance when applied to the compressed data. In our previous work, deep learning compression‐based genomic prediction (DeepCGP) addresses the high‐dimensional data issue by applying autoencoders and RF regression for compression‐based genomic prediction (Breiman, 2001; Liaw & Wiener, 2002). DeepCGP successfully reduces data dimensions while retaining moderate prediction performance using RFs regression method (Islam et al., 2023). Another study by Manthena et al. (2022) explored linear dimensionality reduction techniques using randomized algorithms as preprocessing steps for genomic prediction models. These methods effectively reduce the dimensionality of the data while preserving predictive performance. Recent efforts have also explored federated and fuzzy CNN architectures for modeling epistatic interactions in GWAS settings, further broadening the scope of DL in genomic prediction (Wu et al., 2025).

In this study, we developed a DL‐based method called *C*ompression‐based *G*enomic *P*rediction using *Conv*olutional Neural Network (ConvCGP), which utilizes compressed information generated by autoencoders to predict the genetic values of a target trait. The key contributions of this work are as follows: First, we demonstrate how high‐dimensional genome‐wide polymorphism data can be effectively compressed using autoencoders. Second, we show how the compressed data can be applied to predict genetic values using CNN. Although CNNs have been previously used in genomic prediction, our contribution lies in integrating them with unsupervised, nonlinear compression to form a fully trainable pipeline. This allows the model to capture local dependencies within a compact representation of genomic data, an aspect underexplored in prior work. To assess the performance of ConvCGP, we employed three genome datasets: Cornell‐IR LD Rice Array (C7AIR), with 7098 single‐nucleotide polymorphisms (SNPs); high‐density rice array (HDRA), with 700,000 SNPs; and Maize GSTP004, with approximately 11.7 million SNPs. Our results showed that ConvCGP accurately predicted the genetic values using compressed genome‐wide polymorphism data, achieving performance comparable to predictions made using the original, uncompressed data. In addition, comparison with other methods demonstrated the potential of DL regression techniques in genomic prediction, as ConvCGP maintained high accuracy and outperformed alternative approaches even under extreme compression.

**Core Ideas**

- The rapid growth of genome‐wide data increases computation time for trait prediction in breeding programs.
- Autoencoders compress high‐dimensional data to reduce computational load.
- Convolutional neural networks (CNNs) improve prediction accuracy when trained on compressed genotype data.
- The combined model outperforms standard machine learning and principal component analysis (PCA)‐based methods.
- This method improves efficiency in large‐scale crop genomic prediction tasks.

## 2 MATERIALS AND METHODS

### 2.1 Datasets and data preprocessing

In this study, we used three datasets of varying sizes to illustrate the broad applicability of our models. We assessed the models’ performance by measuring how accurately compressed genome‐wide polymorphism data could predict genetic values of a target trait using a DL‐based regression method. Our aim was to achieve prediction performance comparable to that in models using the original data, while also surpassing other state‐of‐the‐art approaches.

*C7AIR*: The first dataset, C7AIR (Morales et al., 2020), is a second‐generation SNP array containing 189 rice accessions for 7098 markers from the Rice Diversity project. These accessions carried estimated genetic values for plant height (PH).

*HDRA*: The second dataset, HDRA (McCouch et al., 2016), consists of 1568 diverse inbred rice varieties with 700,000 SNPs. Genotypic averages of 34 traits were estimated for 388 lines (Zhao et al., 2011), though some records were missing. We exclude 29 genotypes with 10 or more missing data points, leaving 359 lines across 18 traits for analysis. The genotype data were formatted as a bed matrix derived from a VCF format, where each entry was scored as 0, 1, or 2 to represent the genotype at each SNP: 0 for homozygous reference allele, 2 for homozygous alternate allele, and 1 for a heterozygous state (combinations of different nucleotides such as AT, AC, AG, TC, TG, and CG). Given that all the accessions were inbred lines, expected to be homozygous at most loci, we treated 1 as a missing value and converted 0 and 2 to categorical values (A, C, G, and T). The converted data were saved in CSV format. We used the “gaston” package (Perdry & Dandine‐Roulland, 2018) in R for this conversion.

*Maize GSTP004*: The third dataset used in this study is Maize GSTP004, which consists of 1404 inbred lines genotyped at approximately 11.7 million SNP markers (Liu et al., 2020). For our analysis, we selected a subset of 400 lines evaluated across 20 agronomic traits. Genotype data were processed following the same procedure as described for the HDRA dataset. This dataset provides a high‐resolution genomic resource for dissecting the genetic architecture of complex traits in maize, and has been widely used for genomic prediction and association studies.

We preprocessed the categorical values (A, C, G, and T) in all three datasets by applying one‐hot encoding. Each genome was encoded into one‐hot encoding using a four‐bit coding scheme, where **x**∈ R*^d^* ×4, with *d* representing the length of the genome sequence. The nucleotides “A,” “C,” “G,” and “T” were encoded by “1000,” “0100,” “0010,” and “0001,” respectively. The C7AIR, HDRA, and Maize GSTP004 dataset has ∼13%, ∼10%, and ∼5% missing genotypes, respectively. Therefore, we encoded the missing values “*N*” by “0000.” This encoding ensures that missing data are neutral and do not introduce bias during training. Importantly, we did not apply explicit imputation; instead, missing values are implicitly handled and reconstructed during the autoencoder compression phase as the model learns to capture correlations among neighboring SNPs (Islam et al., 2021).

After one‐hot encoding the raw data, the dimensions of the C7AIR, HDRA, and Maize GSTP004 data became 189 × 28,392, 1568 × 2,800,000, and 400 × 46,751,236, respectively. Because of the high dimensionality of the input data, we applied a data splitting technique to reduce computational time. Using the hsplit function of NumPy, the one‐hot encoded arrays were split along the feature (column) dimension (axis = 1) into smaller chunks. For C7AIR, each split resulted in data of size 189 × 28, while for HDRA, each split was 1568 × 28, creating input layers with 28 neurons for each network. For Maize GSTP004, the one‐hot encoded data were split into segments of 400 × 112, resulting in input layers with 112 neurons per network. Consequently, 1,014 autoencoder networks were used for C7AIR, 100,000 for HDRA, and 417,418 for Maize GSTP004.

### 2.2 Overview of ConvCGP

**FIGURE 1:**
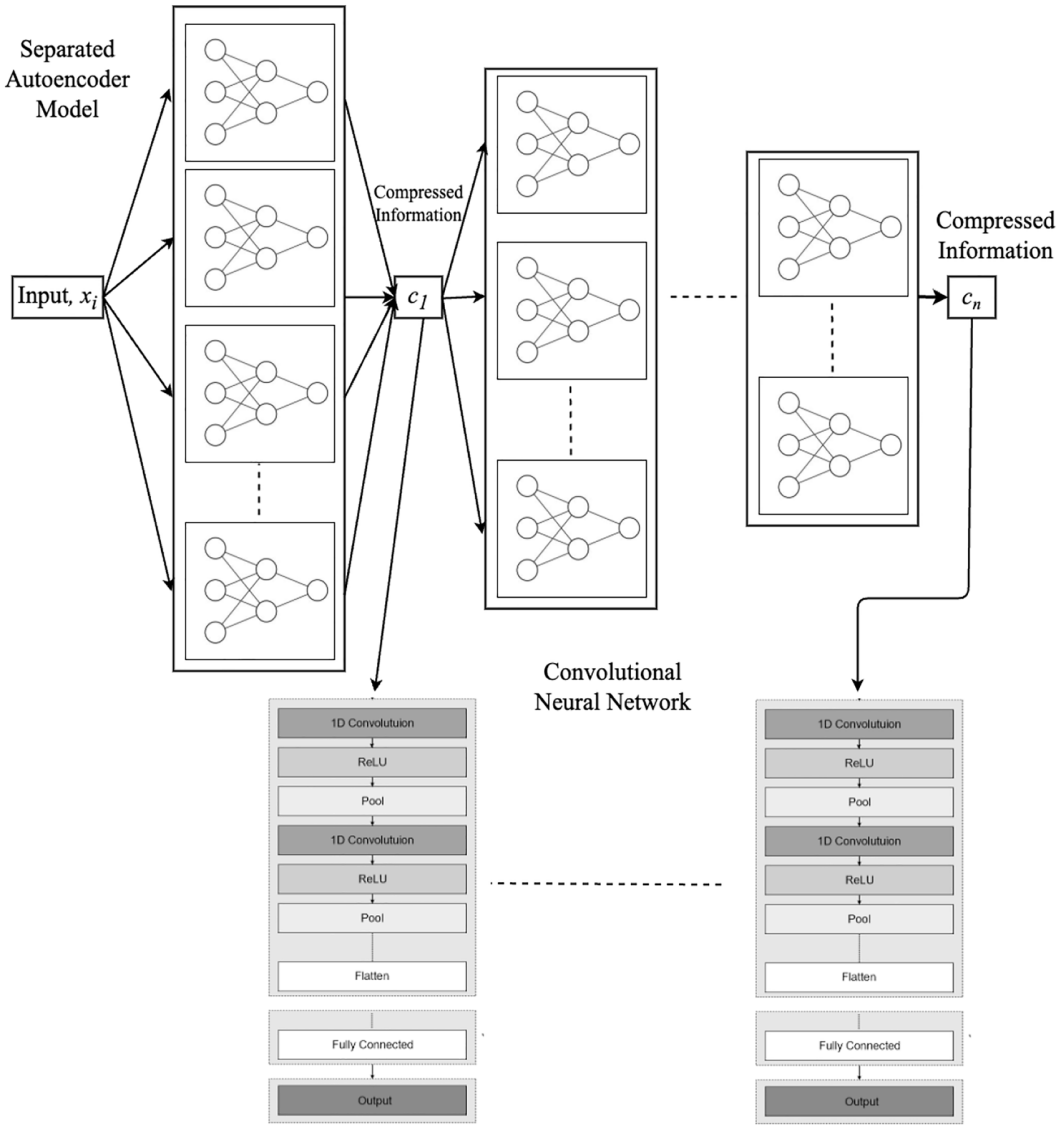
ConvCGP (*C*ompression‐based *G*enomic *P*rediction using *Conv*olutional Neural Network) architecture. The system consists of autoencoder models and a prediction model (i.e., convolutional neural network [CNN]). The autoencoder model compresses the genome‐wide polymorphism data, and the prediction model (CNN) predicts the phenotypes of traits based on compressed genome‐wide polymorphism information.

ConvCGP is a deep‐learning approach designed to predict genetic values by leveraging compressed genomic data (Figure 1). The key innovation of ConvCGP lies in its ability to efficiently reduce the dimensionality of genome‐wide polymorphism data using an autoencoder, followed by prediction through a CNN. While hybrid autoencoder–CNN architectures have been applied in other scientific domains for feature extraction, classification, or generative modeling (Bachay & Abdulameer, 2022; Draizen et al., 2024), these studies do not employ autoencoders as a dedicated large‐scale compression step for genome‐wide polymorphism data nor target genomic prediction as the primary objective. In ConvCGP, the autoencoder serves as a feature extraction mechanism, transforming high‐dimensional genomic data into a lower‐dimensional latent space while preserving essential genetic information. This compression step is crucial for handling large‐scale genomic datasets, as it mitigates computational challenges and reduces redundancy in the data. By learning a nonlinear representation, the autoencoder captures complex genetic relationships that may not be evident in traditional dimensionality reduction techniques. Once the genomic data are compressed, the latent representation is fed into a CNN for prediction. The CNNs are particularly well‐suited for identifying patterns in structured data and have been widely applied in various domains, including image processing and genomics. In our method, the CNN processes the compressed features through multiple convolutional layers, allowing it to extract meaningful representations that contribute to accurate prediction of genetic values.

Traditional dimensionality reduction techniques, such as PCA, use linear transformations to reduce the number of features while preserving variance. However, PCA may struggle to capture complex, nonlinear relationships within genomic data. In contrast, our autoencoder‐based approach learns a nonlinear mapping of the data into a lower dimensional latent space, allowing for a richer representation of genetic variations. Unlike PCA, which directly provides reduced‐dimensional features, our approach includes a decoding step that reconstructs the original input, ensuring that essential genomic information is preserved. Nevertheless, for genomic prediction, only the compressed latent representation is used, making it functionally similar to PCA in this context.

### 2.3 Compression model using deep autoencoder

**FIGURE 2:**
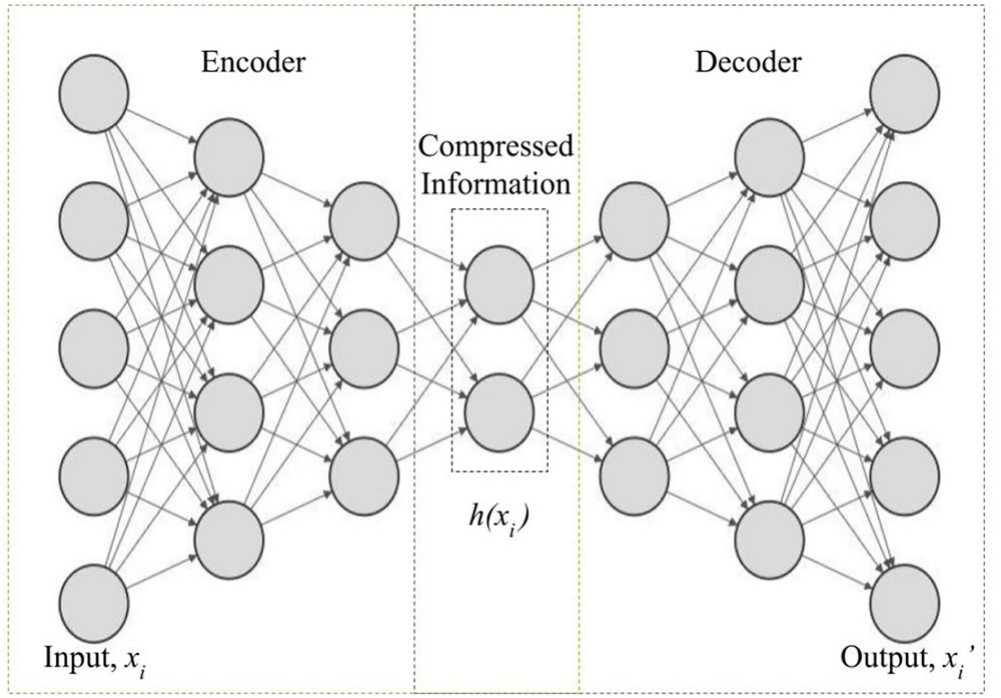
Architecture of a deep autoencoder.

To compress genome‐wide polymorphism data, we utilized a deep autoencoder (Goodfellow et al., 2016; Kramer, 1991) (Figure 2) based on two symmetrical deep belief networks with multiple hidden layers: (i) an encoder network $h(x_{i})$, where $x_{i}\in R^{d}$, which first encodes an input $x_{i}$ into a hidden representation $h{\left(x_{i}\right)}^{\left(l+1\right)}$ as shown in Equation (1), and (ii) a decoder network $x_{i}^{\prime }$, which maps the hidden representation $h{\left(x_{i}\right)}^{\left(l+1\right)}$ back into a reconstruction $x_{i}^{\prime (l)}$ as defined in Equation (2):

(1) $$h{\left(x_{i}\right)}^{\left(l+1\right)}=f\left(W^{\left(l\right)}x_{i}^{\left(l\right)}+b^{\left(l\right)}\right)$$

(2) $$x_{i}^{\prime (l)}=g\left(W^{\prime (l)}h{\left(x_{i}\right)}^{\left(l+1\right)}+b^{\prime (l)}\right)$$

Here, f is the encoding activation function, $W^{\left(l\right)}$ is the encoding weight matrix, $b^{\left(l\right)}$ is the encoding bias vector, g is the decoding activation function, $W^{\prime }$ is the decoding matrix, and $b^{\prime }$ is the decoding bias vector from the l th layer to the (l+1) th hidden layer.

The activation function used for each layer, except the middle and decoder layers, is rectified linear unit (ReLU) (Patterson & Gibson, 2017), which scales negative output value to zero:

(3) $$\mathrm{ReLU}\left(z\right)=\max\left(0,\;z\right)$$

For the middle and decoder layers, the activation function is a “sigmoid” (Patterson & Gibson, 2017), which scales the output to the range [0, 1]:

(4) $$\sigma \left(z\right)=\frac{1}{1+e^{-z}}$$

The reconstruction error was calculated as mean squared error (MSE) function:

(5) $$\mathrm{MSE}\left(x,\;x^{\prime }\right)=\frac{1}{n}\sum_{i=1}^{n}{\left|\left|x_{i}\;-{x^{\prime }}_{i}\right|\right|}^{2}$$

Here, $x_{i}$ and 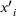 are the measured and predicted values, respectively, and n is the number of samples with i∈[1,n].

An autoencoder model was utilized to compress the genome‐wide polymorphism data. Each dataset was split into training (60%), testing (20%), and validation (20%) sets using the “train_test_split” function of scikit‐learn. To optimize the performance of the compression model, we employed a “KerasRegressor” wrapper from Keras to tune hyperparameters (Table 1) via “RandomizedSearchCV” of scikit‐learn. For the C7AIR dataset, hyperparameter tuning was performed on the entire dataset because of its smaller dimensionality. In contrast, for the HDRA dataset, tuning was performed on a small subset of the training data, specifically the first 1000 splits of data, each containing 1568 × 28 samples. For the Maize GSTP004 dataset, tuning was conducted using 100,000 splits of data, each consisting of 400 × 112 samples.

**TABLE 1:** Hyperparameters determined for the C7AIR (Cornell‐IR LD Rice Array), HDRA (high‐density rice array), and Maize GSTP004 datasets. The learning rate, batch size, and loss function hyperparameters were tuned by exploring values slightly above and below the default range of TensorFlow.

Datasets	Compression level	Neurons	Compressed dimension	Batch size	Epochs
C7AIR (189, 7098)	Compress_1 (57%)	28, 14, 7, 3	(189, 3042)	52	200
	Compress_2 (94%)	36, 28, 10, 5	(189, 425)	32	200
HDRA (1568, 700,000)	Compress_1 (57%)	28, 14, 7, 3	(1568, 300,000)	52	200
	Compress_2 (93%)	30, 15, 5	(1568, 50,000)	32	100
	Compress_3 (98%)	25, 14, 5	(1568, 10,000)	32	150
Maize GSTP004 (400, 11,687,809)	Compress_1 (50%)	112, 84, 56, 14	(400, 5,843,852)	32	50
	Compress_2 (93.75%)	112, 84, 56, 14	(400, 730,422)	32	100
	Compress_3 (99.2%)	112, 84, 56, 14	(400, 91,252)	64	200

*Note*: The optimum learning rate for the adaptive moment estimation (Adam) optimizer was selected from a logarithmic scale of commonly used values: {0.01, 0.001, 0.0001}. Batch sizes of {16, 32, 52, 64} were tested, and the optimal loss was determined by comparing three different loss functions: mean squared error, binary cross‐entropy, and mean absolute error. For each dataset, the compression ratio was computed as (1−*ℎ*/*𝑥*) × 100, where *𝑥* is the original input dimensionality and *ℎ* is the latent dimensionality after compression.

Although the architecture of the autoencoders has been reported in our previous work (Islam et al., 2023) for C7AIR and HDRA datasets, we have described it here for the sake of completeness. For the C7AIR genotype data, the selected model consisted of three hidden layers in both the encoder and decoder networks. In Compress_1, the input layer of a network has 28 nodes, followed by 14 and 7 nodes in the hidden layers, with a code size of 3, which represents the dimensionality of the latent compressed representation. The output from Compress_1 was used as input for Compress_2, which had an input layer of 36 nodes, hidden layers with 28 and 10 nodes, and a code size of 5. Both compressions were trained using the adaptive moment estimation (Adam) optimizer with a learning rate of 0.001. ReLU activation was applied to all layers except the middle and last layers, where sigmoid activation was used. The model was trained with MSE loss, using the mini‐batch size of 52 for Compress_1 and 32 for Compress_2, with 200 epochs for both.

For the HDRA genotype data, the architecture was similar but adapted to the higher dimensionality of the dataset. Compress_1 had layers with [28, 14, 7, 3] nodes, Compress_2 had [30, 15, 5] nodes, and Compress_3 had [25, 14, 5] nodes. Compress_1 was trained with 200 epochs and a batch size of 52, Compress_2 with 100 epochs and a batch size of 32, and Compress_3 with 150 epochs and a batch size of 32. Other parameters were identical to those for the C7AIR data. For the Maize GSTP004 dataset, three levels of compression were applied using an input chunk size of 112. The architecture for all compression stages consisted of layers with [112, 84, 56, 14] nodes. Compress_1 reduced the dimensionality by 50% and was trained for 50 epochs with a batch size of 32. Compress_2 achieved 93.75% compression with 100 epochs and a batch size of 32, while Compress_3 reached 99.2% compression with 200 epochs and a batch size of 64.

The compression model was implemented using Keras functional application programming interface [41], built on TensorFlow in Python. For the C7AIR data, compression levels were Compress_1 (57%), and Compress_2 (94%), meaning compress_1 reduced the data size to 43% of the original, while Compress_2 reduced it to 6%. Similarly, for the HDRA data, Compress_1 (57%), Compress_2 (93%), and Compress_3 (98%) were applied, reducing the data size to 43%, 7%, and 2% of the original, respectively. For the Maize GSTP004 dataset, compression levels were Compress_1 (50%), Compress_2 (93.75%), and Compress_3 (99.2%), corresponding to reductions to 50%, 6.25%, and 0.8% of the original size, respectively. The MSE loss for the C7AIR genotype data were 0.051 for Compress_1 and 0.039 for Compress_2. Similarly, for the HDRA genotype data, the MSE losses remained low, with values of 0.0156 for Compress_1, 0.0749 for Compress_2, and 0.0911 for Compress_3, indicating that high‐quality compression was achieved with minimal loss. For the Maize GSTP004 dataset, the MSE losses were 0.030 for Compress_1, 0.015 for Compress_2, and 0.012 for Compress_3, further confirming stable training and effective compression across different levels.

### 2.4 Prediction model using CNN

**FIGURE 3:**
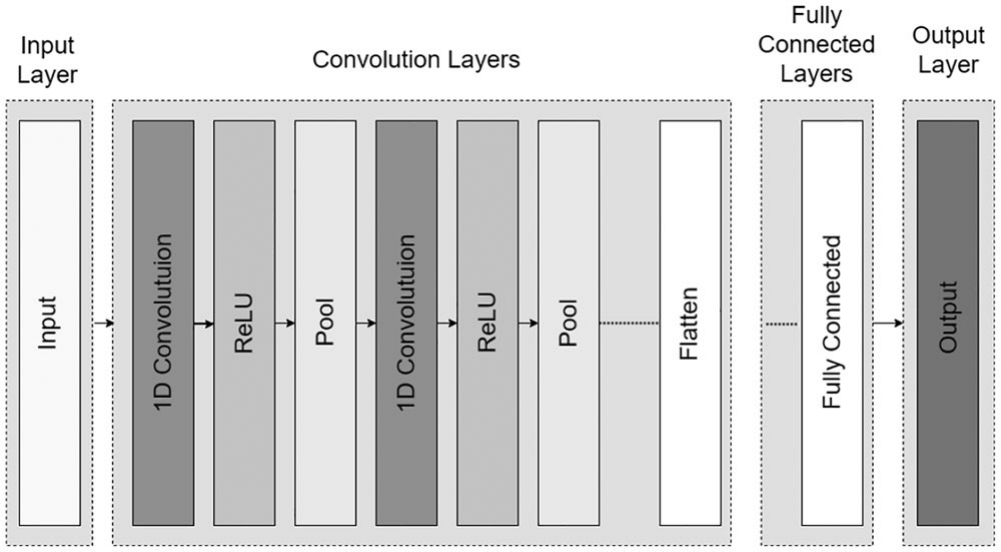
Basic architecture of one‐dimensional (1D) convolutional neural network (CNN).

The CNN architecture (Figure 3) was designed to handle input variables distributed in spatial patterns, such as one‐dimensional (1D) (e.g., SNPs or text) or 2‐D or three‐dimensional (e.g., images). CNNs are neural networks that learn higher order features by using convolution operations in place of full matrix multiplications in the hidden layers (Goodfellow et al., 2016). A typical CNN comprises both dense, fully connected (FC) layers and convolutional layers. In each convolutional layer, the input is processed using learned weight matrices called kernels (or filters), which slide across the input with specified kernel sizes (e.g., a 3 × 3 window) and strides. Each kernel acts somewhat like a neuron's weight vector in an MLP, but instead of connecting to the entire input, it is shared across spatial (or sequential) positions, enabling local feature detection and translation invariance. In the discrete setting for CNNs, the convolution operation is expressed as a finite sum (Sandhu et al., 2020):

(6) $$s\left(t\right)=\left(\;f\times \;k\right)\left(t\right)=\sum_{x=0}^{n-1}k\left(x\right)f\left(t-x\right)\;$$

Here, k represents the kernel (or filter), and the convolution means applying this filter to the input f to produce an output signal s(t). By sliding the kernel along the chromosome, the model can detect local patterns of correlation between nearby variants (linkage disequilibrium). Thus, the convolutional filters can capture these local dependency structures along the chromosome.

To generate the output, an activation function is applied after each convolution. A “pooling” operation is typically used to smooth the result by taking the mean, maximum, or minimum of the kernel outputs at successive positions and merging them. One key advantage of convolutional networks is their ability to reduce the number of parameters to be estimated, while also featuring sparse interactions and translation equivariance. CNNs consist of three main types of layers: convolutional layers, pooling layers, and FC layers (Goodfellow et al., 2016). A CNN architecture is constructed by stacking these layers. Additionally, two important components, the dropout layer and activation function, play a critical role in defining the performance of the model.

The proposed CNN architecture is designed to process 1D input data for a regression task. It begins with four convolutional layers, with filter sizes of 32, 64, 96, and 256, and kernel sizes of 3, 7, 5, and 5, respectively. All convolutional layers use a stride of 1, meaning the convolution window moves one unit at a time. Each convolutional layer is followed by a LeakyReLU activation function (with *α* = 0.1) and BatchNormalization to improve stability and accelerate convergence. MaxPooling1D layers with a pool size of 2 are applied after each convolutional layer to reduce spatial dimensions and indirectly prevent overfitting (e.g., applying MaxPool operations to the CNN's feature map is a form of implicit regularization). To further enhance generalization and mitigate overfitting, dropout layers, with rates of 0.4, 0.4, 0.3, and 0.3, are placed after each BatchNormalization layer to further mitigate overfitting by randomly dropping neurons during training. The model then transitions to two FC layers with 256 and 224 units, each followed by LeakyReLU activation and BatchNormalization. The final output layer is a dense layer with a single unit, providing the regression prediction. L2 regularization with *λ* = 0.01 is applied to the dense layers to prevent overfitting. This architecture integrates convolutional, activation, normalization, pooling, and dropout layers to process and predict from 1D data inputs efficiently. For optimization, we used the Adam optimizer with a learning rate of 0.001, and the model was compiled with MSE loss. The training process incorporated early stopping (patience = 50) to prevent overfitting and improve convergence.

For genetic value prediction using CNN, we used the same two datasets, C7AIR and HDRA, as in our DL compression‐based prediction model. The CNN was applied to both the original uncompressed data and the various levels of compressed data. The estimated genetic values of a target trait, with missing entries omitted, were arranged in the same order as the original and compressed datasets. We divided the datasets into two subsets using the “train_test_split” function of scikit‐learn: 80% for training and 20% for validation. The model was trained on the training subset, while performance was evaluated at regular intervals on the validation subset. In the following section, we outline the process of determining the optimal parameters for training the CNN model.

### 2.5 Determination of hyper‐parameters

Hyperparameter tuning is crucial for optimizing CNN models, given the wide range of possible parameter combinations. In this study, we used the Hyperband tuner for efficient hyperparameter optimization, aiming to minimize the MSE across all evaluated traits. The model was implemented using Keras, incorporating layers such as Conv1D, LeakyReLU, BatchNormalization, MaxPooling1D, Flatten, Dense, and Dropout to reduce overfitting. Hyperparameter optimization was performed using the Hyperband tuner, which iteratively adjusted key parameters, including the number of filters, kernel sizes, dropout rates, the number of convolutional layers, dense layer units, and learning rates. The search space ranged from filters (32–256), kernel sizes (3, 5, and 7), dropout rates (0.3–0.7), dense layer units (256–512 and 128–256), and learning rates (1e‐4 to 1e‐2). The data were divided into training and validation sets, with callbacks like EarlyStopping and ReduceLROnPlateau used to prevent overfitting and ensure efficient training. The optimal hyperparameters were selected based on the lowest validation loss; these parameters—such as specific filter sizes, kernel sizes, dropout rates, and learning rates—were used to build the final CNN model for genetic value prediction.

The final selected hyperparameters were consistent across datasets: the Adam optimizer with a learning rate of 0.001, MSE loss function, and a batch size of 64. For the C7AIR dataset, Compress_1 and Compress_2 were trained for 200 epochs each. For the HDRA dataset, Compress_1, Compress_2, and Compress_3 were trained for 400 epochs each. The Maize GSTP004 dataset used the same hyperparameters as HDRA, with training also conducted for 400 epochs per compression level.

## 3 RESULTS

### 3.1 Prediction of genetic values based on the compressed data using CNN

**FIGURE 4:**
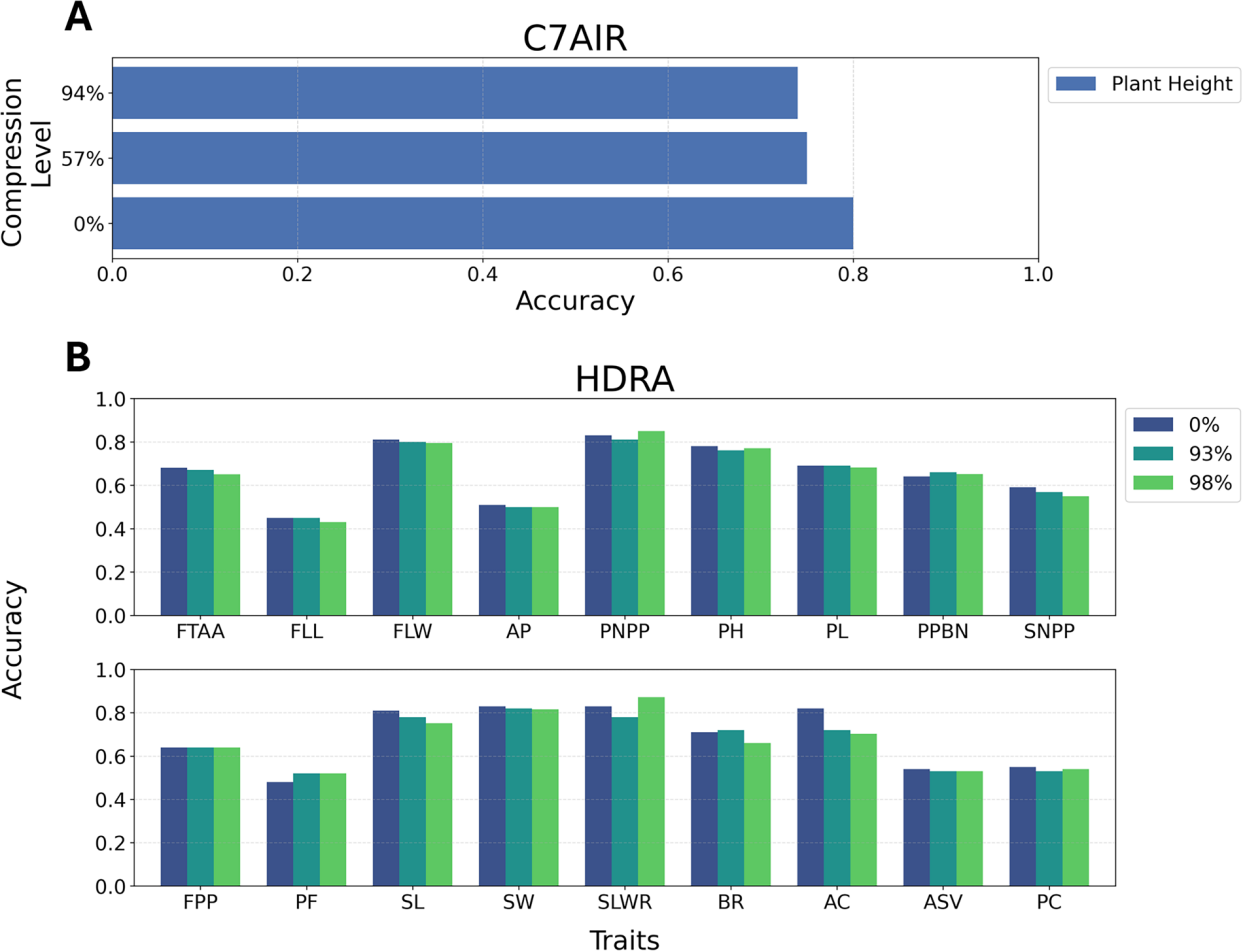
ConvCGP (*C*ompression‐based *G*enomic *P*rediction using *Conv*olutional Neural Network) prediction accuracy for (A) C7AIR (Cornell‐IR LD Rice Array) and (B) HDRA (high‐density rice array) dataset. FTAA, Flowering time at Arkansas; FLL, Flag leaf length; FLW, Flag leaf width; AP, Awn presence; PNPP, Panicle number per plant; PH, Plant height; PL, Panicle length; PPBN, Primary panicle branch number; SNPP, Seed number per panicle; FPP, Florets per panicle; PF, Panicle fertility; SL, Seed length; SW, Seed width; SLWR, Seed length width ratio; BR, Blast resistance; AC, Amylose content; ASV, Alkali spreading value; PC, Protein content.

To evaluate the predictive accuracy of the CNN model and address the impact of data compression, we applied CNN models to the compressed datasets. Figure 4 illustrates the prediction accuracy of CNN across various compression levels for the C7AIR and HDRA datasets. Figure 4A shows the performance of CNN for PH in the C7AIR datasets at three different compression levels. At 0% compression (uncompressed data), the model achieved an 80% accuracy rate. As compression increased to 57%, the accuracy decreased slightly to 75%, and further dropped to 74% at 94% compression. Despite the reduction in data, the CNN maintained a relatively stable and high level of accuracy, demonstrating its robustness in handling compressed genomic data for the prediction.

Figure 4B focuses on the HDRA dataset, highlighting traits that maintain high prediction accuracy even after substantial compression. Notably, traits such as flag leaf width (FLW), panicle number per plant (PNPP), seed width (SW), and blast resistance (BR) consistently show strong performance across all compression levels. These traits exhibit minimal changes in accuracy compared to predictions from the uncompressed data, demonstrating the ability of CNN to preserve prediction quality despite substantial reduction in data dimension. In contrast, even for traits such as protein content (PC) and amylose content (AC), which are typically more challenging to predict because of their complex genetic architecture, ConvCGP maintained reasonably high accuracy under strong compression. This demonstrates the capacity of the model to retain predictive signals even for traits with subtle genetic effects, underscoring its robustness and broad applicability across diverse trait types. Other traits, including plant height (PH), seed number per panicle (SNPP), and seed length (SL), also maintain stable accuracy, showcasing the effectiveness of CNN in preserving predictive performance across various traits. Even at high compression levels, the CNN model outperforms other prediction methods, reaffirming its robustness and adaptability in handling compressed genomic data across a wide range of traits. This suggests that CNNs are highly suitable for predictive modeling in scenarios where data storage or computational resources are limited, as they can still deliver high‐quality predictions from significantly compressed data.

The prediction times for the ConvCGP method across the C7AIR and HDRA datasets show a significant reduction when using compressed data. For the C7AIR dataset, the training time for prediction decreased significantly as compression levels increased. At 0% compression, ConvCGP took 2 min and 50 s, while at 57% compression, the time dropped to 53 s. When data were compressed further to 94%, the prediction time was reduced to a mere 14 s. This trend is consistent in the HDRA dataset: starting at 0% compression, ConvCGP required 1 day, 1 h, and 6 min on average per trait prediction. However, with 93% compression, this time decreased to 54 min and 45 s, and further compression to 98% brought the prediction time down to 39 min and 10 s. These results illustrate the effectiveness of data compression in improving computational efficiency. Even at high compression levels, the quality of predictions remains intact, as indicated by the high levels of retained information across both datasets. Thus, applying data compression prior to prediction not only accelerates processing time but also ensures that essential information is preserved, facilitating faster and equally reliable predictions. Importantly, once ConvCGP is trained, the saved model can be reused for predictions in just a few seconds, thereby saving both computational time and storage, which underscores its practicality for large‐scale genomic prediction tasks.

All experiments in this study were conducted on a PC with an Intel(R) Core (TM) i9‐10980XE, 3.00 GHz CPU, 128 GB RAM, GPU RTX 3090, and a 64‐bit Windows 10 pro operating system.

### 3.2 Comparing prediction performance with other methods

To rigorously evaluate our method ConvCGP, we conducted a comparative analysis against several baseline models commonly used in genomic prediction, including GBLUP, Lasso regression, support vector machines (SVM), two recent approaches based on kinship matrices, and principal component scores (PCs), as well as our previous framework, DeepCGP. For the kinship and PC‐based methods, dimensionality reduction was first applied, followed by a CNN to assess how well traditional reduction techniques preserve predictive information. In contrast, SVM, Lasso, and DeepCGP were applied to the compressed representations produced by the autoencoder, allowing us to isolate the contribution of the CNN in the ConvCGP pipeline.

Kinship matrices (Nazzicari & Biscarini, 2022), such as the realized additive (𝐾_A_), dominance (𝐾_D_), additive‐by‐additive (K_AA_), additive‐by‐dominance (K_AD_), and dominance‐by‐dominance (𝐾_𝐷𝐷_), represent genetic relationships. SNPs are filtered based on minor allele frequency, and the five genetic relationships (K_A_, K_D_, K_AA_, K_AD_, K_DD_) yield 15 kinship matrices. These kinship matrices condensed representations of genetic relatedness derived from genome‐wide marker genotype data, reduce input dimensionality, and when stacked within a 2D‐CNN, leverage genetic similarities to improve predictions for continuous genetic values of traits. In our datasets, we only used K_A_ and K_AA_ matrices, as the HDRA dataset contains few heterozygous genotypes. Since the dominance effect primarily affects heterozygous genotypes, its inclusion had minimal impact on the overall results. Another approach using PCs involves PCA to reduce input dimensions, followed by multimodal DL techniques. These techniques have shown comparable prediction accuracy between DL and GBLUP (Kick et al., 2023). The PCs captured 31% of the variance with eight PCs, 50% with 50 PCs, and over 99% with 1725 PCs. For the PCs analysis, we removed one negative eigenvalue from the HDRA data and reduced the dimensionality. In addition to these kinship matrices and PC‐based approaches, we compared our method with GBLUP, Lasso, SVM, and our previous work, DeepCGP, a compression‐driven predictive model that uses DL autoencoders for data compression and RF regression for prediction (Islam et al., 2023).

**FIGURE 5:**
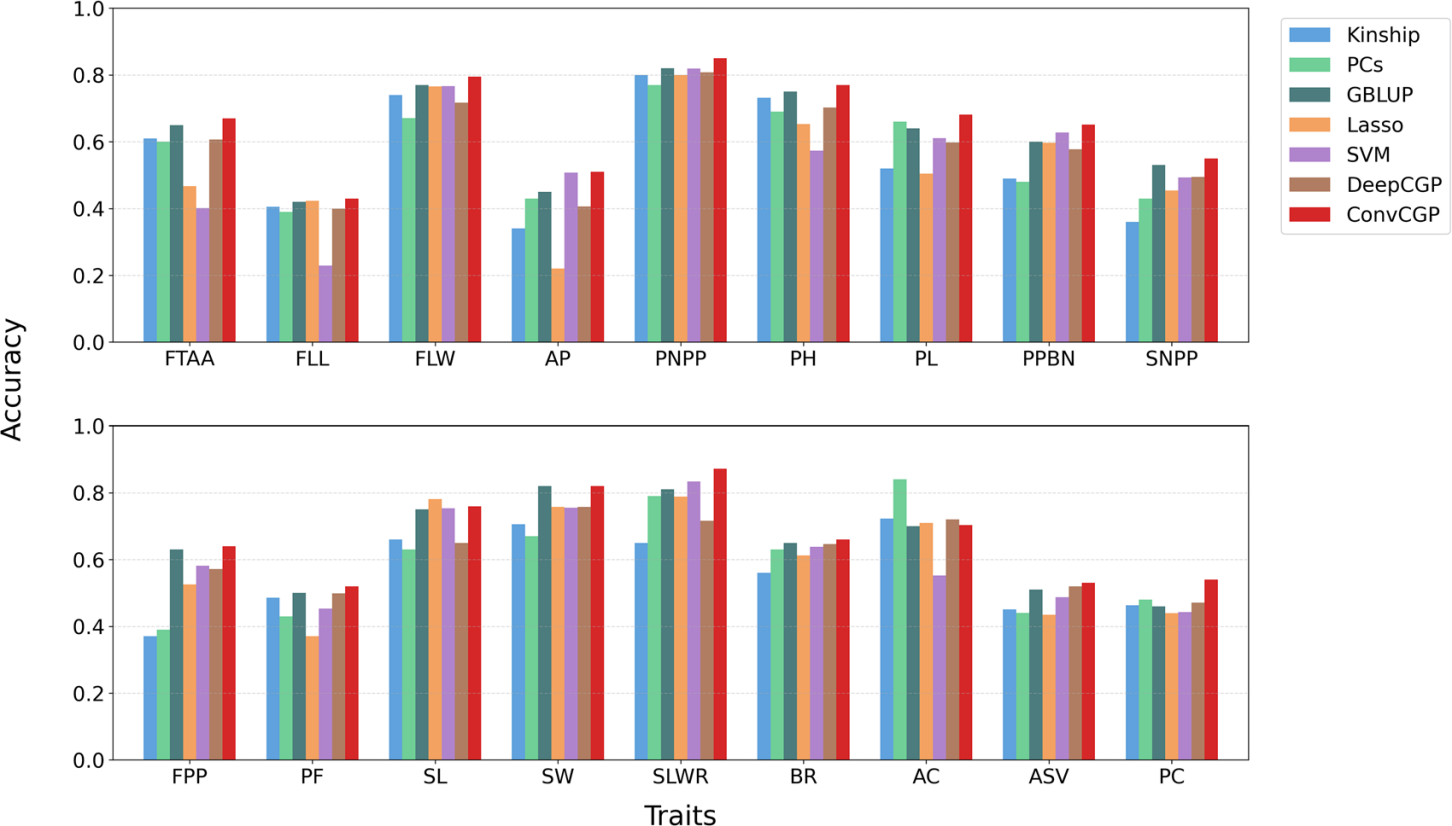
This figure illustrates the prediction accuracy of seven models—Kinship, PCs (principal component scores), GBLUP (genomic best linear unbiased prediction), Lasso (least absolute shrinkage and selection operator), SVM (support vector machine), DeepCGP (deep learning compression‐based genomic prediction), and ConvCGP (*C*ompression‐based *G*enomic *P*rediction using *Conv*olutional Neural Network)—across 18 traits using a 98% compressed version (i.e., 2% of the original HDRA [high‐density rice array] dataset). The vertical axis represents prediction accuracy, while the horizontal axis lists the traits. ConvCGP consistently outperforms the other models for most traits, demonstrating its ability to handle highly compressed data effectively. Notably, for traits such as flowering time at arkansas (FTAA), panicle number per plant (PNPP), plant height (PH), seed length‐width ratio (SLWR), and protein content (PC), ConvCGP achieves significantly higher accuracy compared to Kinship, PCs, GBLUP, Lasso, SVM and DeepCGP. ConvCGP also excels in predicting key quality traits like blast resistance (BR), where other models struggle. These results indicate that ConvCGP is particularly skilled at capturing intricate genetic patterns, even when the dataset has been compressed to retain only 2% of the original information.

Figure 5 illustrates the prediction accuracy of seven models—Kinship, PCs, GBLUP, Lasso, SVM, DeepCGP, and ConvCGP—across 18 traits using a 98% compressed version i.e 2% of the original HDRA dataset. The vertical axis represents prediction accuracy, while the horizontal axis lists the traits. ConvCGP consistently outperforms the other models for most traits, demonstrating its ability to handle highly compressed data effectively. Notably, for traits such as flowering time at arkansas (FTAA), panicle number per plant (PNPP), plant height (PH), seed length‐width ratio (SLWR), and protein content (PC), ConvCGP achieves significantly higher accuracy compared to Kinship, PCs, GBLUP, Lasso, SVM and DeepCGP. ConvCGP also excels in predicting key quality traits like blast resistance (BR), where other models struggle. These results indicate that ConvCGP is particularly skilled at capturing intricate genetic patterns, even when the dataset has been compressed to retain only 2% of the original information.

**FIGURE 6:**
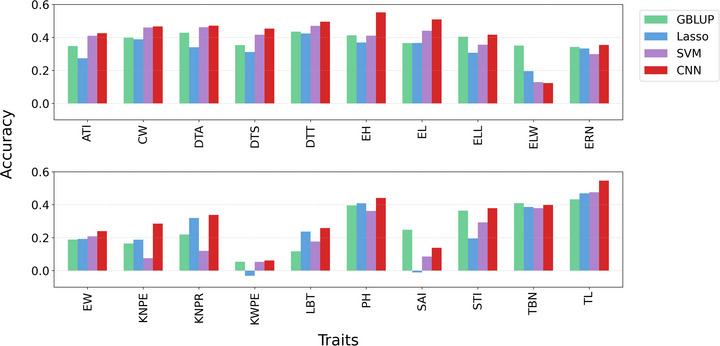
Prediction accuracy of GBLUP (genomic best linear unbiased prediction), Lasso, SVM (support vector machine), and ConvCGP (*C*ompression‐based *G*enomic *P*rediction using *Conv*olutional Neural Network) for the Maize dataset. ATI, Anther‐tassel interval; CW, Cob weight; DTA, Days to anther; DTS, Days to silk; DTT, Days to tassel; EH, Ear height; EL, Ear length; ELL, Ear leaf length; ELW, Ear leaf width; ERN, Ear row number; EW, Ear weight; KNPE, Kernel number per ear; KNPR, Kernel number per row; KWPE, Kernel weight per ear; LBT, Length of barren tip; PH, Plant height; SAI, Silk‐anther interval; STI, Silk‐tassel interval; TBN, Tassel branch number; TL, Tassel length.

In addition to rice, we further validated our approach on the Maize GSTP004 dataset (400 inbred lines, 20 traits). Figure 6 shows the prediction performance of GBLUP, Lasso, SVM, and ConvCGP after autoencoder‐based compression. Consistent with the HDRA results, ConvCGP achieves the highest accuracy across the majority of traits, including key agronomic features such as tassel length (TL), ear height (EH), and plant height (PH). Although GBLUP, Lasso, and SVM yielded only moderate accuracies, ConvCGP consistently captured complex nonlinear patterns, underscoring the strength of DL in modeling intricate genomic architectures. Notably, this superior performance was obtained under extreme compression, retaining only 2% of the original data, yet ConvCGP still delivered robust and accurate predictions across diverse traits and crops. Importantly, ConvCGP also demonstrated competitive efficiency: Its trained prediction time per trait on the maize dataset was 8 s, comparable to SVM (∼4 s) and even faster than Lasso (∼20 s) and GBLUP (∼3 min). Thus, ConvCGP combines state‐of‐the‐art predictive accuracy with practical runtime efficiency, establishing it as a more powerful and scalable solution for genomic prediction than traditional methods.

The improvement in prediction accuracy is primarily attributed to the ability of autoencoders to capture nonlinear relationships in the data, rather than the specific choice of the downstream prediction model. The advantage of ConvCGP arises from the combination of nonlinear compression with convolutional layers, which enables the extraction of meaningful patterns from sparse genomic data. For the HDRA dataset, ConvCGP consistently surpasses DeepCGP and traditional approaches, with particularly strong gains for flowering time, PH, and blast resistance. In maize, the same trend holds across multiple traits, confirming that ConvCGP is robust under extreme compression and adaptable across species. Together, these findings provide strong evidence that ConvCGP performs favorably compared with baseline methods and support its potential as a powerful framework for large‐scale genomic prediction even under extreme data compression.

## 4 DISCUSSION

Efficient use of high‐dimensional genome‐wide polymorphism data for plant and animal breeding requires innovative platforms, which significantly reduce the resources required for storage (Islam et al., 2023) and processing. Few studies have focused on reducing the dimensions of genome‐wide polymorphism data while simultaneously predicting genetic values for a target trait. The kinship matrix, PC, and DeepCGP approaches were examined. Although both kinship matrices and PCs successfully reduced dimensionality and enabled predictions from compressed data, their prediction accuracy remained low. Conversely, in our previous study, DeepCGP, which combined DL for compression with machine learning for prediction, outperformed kinship matrices and PC‐based methods. However, we identified the potential to further enhance prediction accuracy by adopting a DL‐based prediction method rather than relying on machine learning for the prediction phase, as in DeepCGP.

In this study, we proposed ConvCGP, a deep‐learning‐based method that improves prediction accuracy by utilizing CNNs to predict from compressed data produced by autoencoders. The compressed data (latent representation) obtained from the autoencoder in ConvCGP is designed to capture non‐linear relationships within the genomic data. These relationships often correspond to biologically meaningful patterns, such as population structure or trait‐associated variants, rather than random noise. Specifically, each autoencoder in ConvCGP compresses adjacent SNPs together, effectively preserving the local genotype patterns of SNPs in linkage disequilibrium. This localized compression ensures that the encoded representation reflects the genetic similarity within specific genome segments, enhancing the biological relevance of the compressed data. Additionally, when multiple layers of autoencoders are stacked for progressive compression, the architecture continues to rely on adjacent genomic regions, maintaining the genetic continuity and similarity of those regions. This approach contrasts with PCA, which compresses data based on global variation patterns, potentially overlooking local genomic structures. Notably, ConvCGP does not rely on GWAS for compression. Although GWAS‐based approaches necessitate trait‐specific marker selection, ConvCGP produces a shared compressed representation that remains applicable across multiple traits. This trait‐agnostic approach enhances the versatility of ConvCGP, making it a robust tool for modeling complex genomic relationships without bias toward any particular phenotype.

To further highlight the contributions of ConvCGP, we compare it in greater depth with other commonly used genomic prediction methods GBLUP, SVM, and Lasso. Compared to existing approaches, ConvCGP offers a more unified and scalable framework for genomic prediction. PCA is a commonly used linear technique for dimensionality reduction that efficiently captures global variance but may overlook fine‐scale local genomic patterns. Kinship matrix‐based methods provide biologically meaningful representations of genetic similarity; however, they rely on predefined relationship structures and may not fully capture nonlinear dependencies. DeepCGP, our earlier approach, demonstrated that autoencoders can successfully compress high‐dimensional SNP data while preserving predictive signals when paired with machine learning models like RFs. Building on this foundation, ConvCGP advances the compression–prediction pipeline by integrating CNNs directly into the end‐to‐end learning process. This allows the model to jointly learn compressed representations and predictive features, capturing local genomic dependencies more effectively, while maintaining the flexibility and interpretability of DL. Through this integration, ConvCGP further improves prediction accuracy and efficiency, especially under high compression rates.

ConvCGP method was applied to rice and maize dataset by employing CNN architectures with convolutional, pooling, and FC layers to predict the genetic values of traits from compressed genome‐wide polymorphism data. The convolutional layers, consisting of multiple convolution kernels, generated various feature maps from the input representations. Each neuron in the feature map was connected to a specific region in the neighboring neurons of the previous layer. The CNN architecture was constructed by stacking layers, and during training, we optimized the algorithm and selected network parameters. With a relatively shallow network (three or five layers) and carefully tuned parameters, we achieved 99.3% relative accuracy using genome‐wide polymorphism data that had been compressed by 99%, reducing the data size to just 1% of its original size. This level of accuracy was comparable to results using the original data, demonstrating the effectiveness of ConvCGP in predicting genetic values from highly compressed genome‐wide polymorphism data.

We developed a compression‐based genomic prediction model, ConvCGP, using DL, which enhances breeding efficiency while significantly reducing the storage requirements for genome‐wide polymorphism data. The key advantage of using DL for compression lies in its ability to extract meaningful information from the genetic architecture, effectively modeling complex patterns with fewer computational resources compared to conventional approaches. The experimental results are promising, achieving high accuracy of genotypic value prediction while maintaining robustness even with compressed data. Our model allows flexibility in compression levels, which can be adjusted based on storage constraints, the time required to construct a CNN, or accuracy requirements. For example, higher compression levels (e.g., 94–99%) can be adopted in large‐scale screening programs to save computational time, while moderate compression levels (e.g., 50%–70%) may be more suitable for applications demanding higher accuracy, such as precision breeding for complex traits. Additionally, the parameters of ConvCGP, such as CNN depth and regularization, can be fine‐tuned based on trait complexity, data size, or resource availability.

ConvCGP addresses high‐dimensionality challenges by using a scalable architecture with multiple autoencoders in parallel, efficiently managing data while maintaining linear computational complexity. As SNP numbers increase, more autoencoders are added, but each retains a constant computational load. ConvCGP was validated on both rice germplasm and the large‐scale maize GSTP004 dataset, demonstrating robust performance across different crops and trait types. Although these results strongly support its scalability and generalizability, further evaluation on additional plant and animal datasets will help establish its broader applicability. Nonetheless, the benchmarking results in this study provide stronger evidence of the advantage of ConvCGP, particularly in handling complex traits with highly compressed genomic data. In addition, genomic data quality and training population size can affect the performance of ConvCGP. As with most DL models, ConvCGP benefits from large, high‐quality datasets, while sparse or noisy data may reduce compression and prediction effectiveness. Future work research will investigate its applicability across more high‐dimensional datasets to evaluate its robustness in diverse genetic backgrounds. Additionally, we plan to analyze the gradients within the neural network that predict genetic values from high‐dimensional genome‐wide polymorphism data, and explore the potential of DL‐based compression methods to identify important SNP sets. To further enhance the applicability of ConvCGP under different data quality and resource conditions, future extensions may also consider strategies such as transfer learning, hybrid modeling with biological priors, or semi‐supervised learning to mitigate the effects of limited or noisy training data.

## 5 CONCLUSION

In summary, ConvCGP, a novel DL model, was introduced as an innovative solution for compressing high‐dimensional genome‐wide polymorphism data while accurately predicting genetic values from the compressed information. The novelty of ConvCGP lies in its integration of unsupervised, nonlinear compression using autoencoders with CNN‐based supervised learning in a single, end‐to‐end trainable framework, an approach that remains underexplored in existing genomic prediction literature. ConvCGP demonstrates great potential for complex modeling, particularly through its novel application of DL in genomic prediction. By using compressed data as input variables, this approach significantly improves computational efficiency in CNN‐based DL. Additionally, ConvCGP provides a robust solution for handling high‐dimensional genomic data, maintaining high prediction accuracy even with compressed inputs. This was validated not only in rice but also in maize, where ConvCGP consistently outperformed GBLUP, Lasso, and SVM, confirming its adaptability across different crop contexts. It also offers clear advantages in terms of storage efficiency and reduced computational time, making it a promising tool for genomic prediction and selection in plant breeding.

## AUTHOR CONTRIBUTIONS

**Tanzila Raihan**: Conceptualization; data curation; formal analysis; investigation; methodology; software; validation; visualization; writing—original draft; writing—review and editing. **Chyon Hae Kim**: Conceptualization; methodology; project administration; supervision; writing—review and editing. **Hiroyuki Shimono**: Funding acquisition; project administration; supervision; writing—review and editing. **Akio Kimura**: Project administration; supervision; writing—review and editing. **Hiroyoshi Iwata**: Conceptualization; funding acquisition; methodology; project administration; resources; supervision; validation; writing—review and editing.

## CONFLICT OF INTEREST STATEMENT

The authors declare no conflicts of interest.

## Data Availability

Our code is available at https://github.com/tanzilamohita/ConvCGP Data are publicly available at https://iagr.genomics.cn/CropGS
